# Phytochemical Composition, Antibacterial Activity, Modes of Action, and Antibiotic Resistance–Modifying Effects of *Harungana madagascariensis* (Hypericaceae) Against Multidrug-Resistant *Pseudomonas aeruginosa*

**DOI:** 10.1155/sci5/8950117

**Published:** 2025-07-13

**Authors:** Richard Mouozong, Aimé Gabriel Fankam, Varelle Lambou Diffo, Victor Kuete

**Affiliations:** Department of Biochemistry, University of Dschang, Dschang, Cameroon

**Keywords:** antibacterial, antibiotic resistance–modifying effects, *Harungana madagascariensis*, multidrug resistance, phytochemicals, *Pseudomonas aeruginosa*

## Abstract

*Harungana madagascariensis* is a plant used in African traditional medicine to treat a wide range of human diseases, including microbial infections. The aim of this study was to evaluate the phytochemical composition, antibacterial activity, modes of action, and antibiotic resistance-modifying effects of the leaf, bark and root extracts of *H*. *madagascariensis* against multidrug-resistant *Pseudomonas aeruginosa*. The broth microdilution method was used to evaluate the antibacterial activity and antibiotic resistance–modifying effects of extracts. Phytochemical composition of extracts was carried out using known qualitative and quantitative methods. The action of the most active extract was evaluated on the bacteria cell membrane and catalase activity. The phytochemical results indicated that all the extracts contain alkaloids, terpenoids, saponins, phenols, flavonoids, tannins, and anthocyanins. Moreover, *H*. *madagascariensis* leaf extract (HMLE) had the highest phenolic (107.41 ± 9.66 mg GAE/g of extract) and flavonoid (53.67 ± 5.09 mg QE/g of extract) contents. The extracts had a wide range of antibacterial activity, with MICs ranging from 16 to 2048 μg/mL. HMLE identified as the most active extract affected the cytoplasmic membrane integrity and inhibited the catalase activity of *P*. *aeruginosa*. Moreover, HMLE at its subinhibitory concentration (MIC/8) improved the antibiotic activity by 2- to 16-fold. The MICs of tetracycline and doxycycline deceased from 32 to ≤ 2 *μ*g/mL and that of kanamycin from 256 to 32 μg/mL against the tested MDR *P*. *aeruginosa*. In conclusion, this study indicates that extracts from *H. madagascariensis*, particularly from its leaves, could serve as valuable assets in the discovery of new treatment option of infections due to MDR *P*. *aeruginosa*.

## 1. Introduction

The rise of multidrug resistance among bacteria thwarts the treatment of bacterial diseases using the current antibacterial medications, leading to an increase in both morbidity and mortality rates [1]. *Pseudomonas aeruginosa* is a pathogenic bacterium responsible for the majority of infections that occur in the hospital. It causes bacteraemia, wound infection, respiratory and urinary tract infections [2, 3]. *P*. *aeruginosa* resistance to many antibiotics is attributed to its outer membrane low permeability, its active efflux pumps and other resistance mechanisms, such as the generation of beta-lactamases, aminoglycoside-modifying enzymes and quinolone resistance mechanisms [4]. The 2019 report from the Centres for Disease Control and Prevention (CDC) has classified multidrug-resistant (MDR) *P*. *aeruginosa* as a serious threat, contributing to at least 32,600 hospitalisations, 2700 deaths and an estimated $767 million in healthcare expenditures in the United States [5]. As bacteria increasingly acquire MDR traits, there is an urgent need to seek new antimicrobial drugs [6]. This is especially true for MDR *P*. *aeruginosa* that has been classified as “critical” on the World Health Organization's (WHO) priority list of bacterial pathogens for which exploration and development of new antibiotics are in dire need [7]. Plants appear as reservoir of biologically active components. Medicinal plants have been demonstrated to be rich sources of secondary metabolites with different chemical structures and mechanisms of action which may be precious in developing new treatments against microbial infections including those caused by MDR bacteria [8, 9].

*Harungana madagascariensis* Lam. ex Poir (Hypericaceae) is locally known as “Nketto” in Cameroon by the Bamileke tribe or “Aranje” in Nigeria by the Yoruba tribe [10]. The “Harungana” plants are widely distributed in Africa [11]. Over many years, *H*. *madagascariensis* has been used in African traditional medicine to treat a wide range of human diseases, including dysentery, diarrhoea, jaundice, typhoid fever, haemorrhoids, leprosy, gonorrhoea, anaemia, postpartum bleeding, along with skin and heart problems [10, 12–15]. *H*. *madagascariensis* also has a rich phytochemical variability. Compounds including anthranoids, anthraquinones, xanthones, flavonoids, triterpenoids, steroids and alkaloids have been isolated in its different parts [10]. The literature has reported different pharmacological properties of extracts or compounds isolated from *H*. *madagascariensis* among which are antityphoid [14], antioxidant [15], antiprotozoal [16], antimicrobial [17, 18], antisickling [19] and antiproliferative [20] activities. Although extracts from *H*. *madagascariensis* have been reported to have in vitro antimicrobial activity against various pathogens [17], no details on their activity, modes of action and antibiotic resistance–modifying effects have been reported against many MDR *P*. *aeruginosa*. Moreover, the previous studies were not based on root extract. Therefore, this study was designed to investigate the phytochemical composition, antibacterial activity, modes of action and antibiotic resistance–modifying effects of the leaf, bark and root extracts of *H*. *madagascariensis* against MDR *P*. *aeruginosa*.

## 2. Materials and Methods

### 2.1. Plant Materials

The leaves, roots and bark of *H*. *madagascariensis* (Hypericaceae) were harvested in August 2022 at Foréké-Dschang (5°24′18″N, 10°00′57″E), West Cameroon. This plant was identified at the National Herbarium of Cameroon, Yaoundé, under the voucher specimen number 43848/HNC.

### 2.2. Preparation of Extracts

All the collected plant organs were cleaned and air-dried in the absence of UV light. One hundred grams of dried powders of each argan was extracted by 300 mL of 95% methanol for 48 h at room temperature, filtering through Whatman filter paper No. 1, and concentrated at 65°C under reduced pressure on rotary evaporator (BÜCHI R-200) to obtain the crude extracts. Then, the residual methanol was completely evaporated by drying the extracts at 40°C in an oven, and extracts were stored at 4°C until further use.

### 2.3. Bacteria and Culture Conditions

*P*. *aeruginosa* used in this study included one reference strain (ATCC 27853) from American Type Culture Collection, and nine MDR clinical isolates (PA1, PA2, PA3, PA4, PA7, PA9, PA12, PA14 and PA124). Their features were previously reported [21, 22]. The tested bacteria were cultured and maintained on Mueller–Hinton agar (MHA). The inoculum for antibacterial assay, modes of action determination and modulating assays was in Mueller–Hinton broth (MHB).

### 2.4. Determination of the Phytochemical Composition

#### 2.4.1. Preliminary Phytochemical Screening

In order to detect the presence of important secondary metabolite classes in the leaf, bark and root extracts of *H*. *madagascariensis*, the following standard phytochemical screening methods were used: Mayer's test for the detection of alkaloids, Salkowski's test for the detection of terpenoids, Froth's test for the detection of saponins, ferric chloride test for the detection of phenols, lead acetate test for the detection of flavonoids, ferric chloride test for the detection of tannins and HCl test for the detection of anthocyanins [21, 23].

#### 2.4.2. Determination of the Total Phenolic Content (TPC) of Extracts

The TPC of extracts was determined using the Folin–Ciocalteu method [24]. In a test tube, a mixture of 0.2 mL of 2 N Folin–Ciocalteu reagent, 0.02 mL of extract (2 mg/mL) and 0.4 mL of 20% sodium carbonate solution was prepared, then agitated and incubated for 20 min at 40°C in a water bath. After incubation, the absorbance of the mixture was measured at 760 nm, and the results were expressed in milligrammes of gallic acid equivalents per gram of dry extract (mg GAE/g) using a gallic acid standard curve. Each sample was carried out in triplicate.

#### 2.4.3. Determination of Total Flavonoid Content (TFC) of Extracts

The TFC of the tested extracts was assessed using the aluminium trichloride method as previously described [25]. Briefly, 100 μL of the extract (2 mg/mL) was combined with 1.49 mL of distilled water and 30 μL of 5% NaNO_2_. This mixture was incubated for 5 min at room temperature, after which 30 μL of 10% AlCl_3_ was added. Following an additional 6 min incubation, 200 μL of 0.1 M NaOH and 240 μL of distilled water were added to the solution. The mixture was then thoroughly mixed, and the absorbance was measured at 510 nm. The results were expressed in milligrammes of quercetin equivalents per gram of dry extract (mg QE/g), calculated using a quercetin standard curve. Each sample underwent triplicate analysis.

### 2.5. Evaluation of the Antibacterial Activity of Extracts

The microdilution method was employed to assess the minimum inhibitory concentration (MIC) of the plant extracts utilising INT calorimetric assays as previously described [26] with some modifications [27]. Briefly, 100 μL of extracts or reference antibacterial dissolved in DMSO/MHB were serially diluted in a 96-well microplate follows by the addition of 100 μL of bacterial inoculum (2 × 10^6^ CFU/mL) to each well. After 18 h of incubation at 37°C, the MICs of the samples were detected by adding 40 μL of INT (0.2 mg/mL) and incubating at 37°C for 30 min. The final concentrations of extracts and antibiotics varied from 16 to 2048 μg/mL and from 2 to 256 μg/mL, respectively. Imipenem (IMI) served as standard antibacterial. Wells containing only MHB were used as a negative control, while those containing bacterial inoculum served as a growth control. IMI was used as the reference antibiotic. The minimum bactericidal concentration (MBC) of the sample was assessed by subculturing 50 μL of the suspensions from the wells that showed no growth after the MIC test into 150 μL of fresh MHB, followed by reincubation at 37°C for 48 h. The MBC was identified as the minimal concentration of the samples that did not show pink colour after the INT solution was added [27]. Each experiment was carried out in duplicate and repeated three times.

### 2.6. Determination of the Possible Modes of Action of the Most Active Extract

#### 2.6.1. Evaluation of the Effect of Extract on the Cell Membrane Integrity

The effect of *H*. *madagascariensis* leaf extract (HMLE) on the cell membrane integrity of *P*. *aeruginosa* PA2 was assessed as previously described [28] with slight modifications. Briefly, the test bacteria in the exponential growth phase in MHB were centrifuged at 400 × *g* for 15 min, and the pellet was washed twice in phosphate-buffered saline (PBS), pH 7.4. The gathered bacteria collected were resuspended in 10-mM PBS (pH 7.4) and incubated with the HMLE at its MIC and 2xMIC for various durations (0, 3, 6, 9 and 12 h). Vancomycin (VAN) at 32 μg/mL was used as positive control. Following the incubation of each treatment at 37°C for 60 min, the cell suspension was centrifuged at 13, 400 × *g* for 15 min, and the absorbance of each supernatant was recorded at 260 nm using a spectrophotometer (Biobase Bk-D590 Double Beam Scanning UV/Vis, China) and reflected the concentration of the 260-nm absorbing materials released by bacterial cells. Cells that did not contain extract served as control.

#### 2.6.2. Evaluation of the Catalase Inhibitory Activity of Extract

The catalase inhibitory activity of HMLE was carried out as described by Sawant et al. [29]. Fresh colonies of *P*. *aeruginosa* (PA2) were introduced into 5 mL of sterile MHB and incubated at 37°C in a shaker overnight to prepare untreated culture controls. Likewise, fresh colonies of PA2 were cultured in a medium containing 1 mL of HMLE (MIC and 2xMIC) and 4 mL of MHB. One hundred μL of the relevant test samples with an OD of 0.1 was transferred into a test tube. Then, 100 μL of 1% Triton X-100 and 100 μL of 30% (v/v) H_2_O_2_ were introduced into the tube. Once thoroughly mixed, the tubes were kept at room temperature for 5 min, and the stable foam height was measured with a ruler.

### 2.7. Modulation Assay

To evaluate the antibiotic modulation effects of plant extracts, the MICs of antibiotics such as tetracycline (TET), doxycycline (DOX), kanamycin (KAN), streptomycin (STR), VAN, ciprofloxacin (CIP), ceftriaxone (CEF), IMI and ampicillin (AMP) (Sigma-Aldrich) were determined in the presence and absence of extracts at subinhibitory concentration (MIC/8) using the microdilution method [30, 31]. The modulation factor (MF) was determined as the ratio of the MIC of the antibiotic alone to the MIC of antibiotic combined with the extract: MF = (MIC antibiotic)/(MIC antibiotic + extract). A MF ≥ 2 was established as the threshold for the biological significance for antibiotic resistance–modifying effect of the extract [32].

### 2.8. Statistical Analysis

The data reported as mean with the standard deviation (Mean ± SD) from three replicates were analysed using GraphPad Prism for windows, Version 5.0.1. One-way analysis of variance (ANOVA) followed by Tukey's multiple comparison test was used to compare the means, with a probability level of *p* < 0.05 considered significant.

## 3. Results

### 3.1. Phytochemical Composition

#### 3.1.1. Qualitative Phytochemical Content

Phytochemical screening showed that the extracts tested contain all the secondary metabolites sought in this study. These included alkaloids, terpenoids, saponins, phenols, flavonoids, tannins, and anthocyanins (Table 1).

#### 3.1.2. TPC and TFC of Extracts

**Figure 1:**
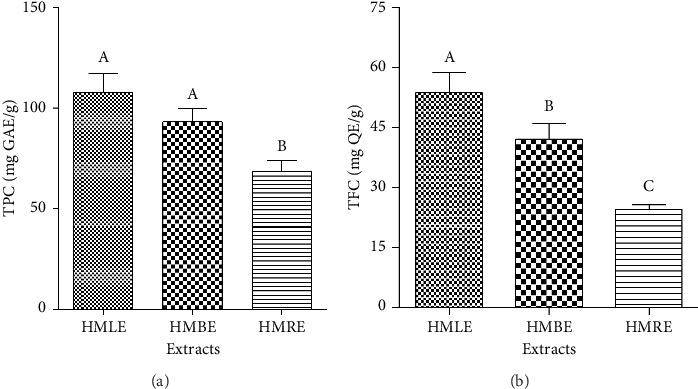
Chemical composition of *H*. *madagascariensis* extracts. (a) Total phenolic content; (b) total flavonoid content. Values followed by different letters are significantly different (*n* = 3, *p* < 0.05). HMLE: *H*. *madagascariensis* leaf extract; HMBE: *H*. *madagascariensis* bark extract; HMRE: *H*. *madagascariensis* root extract; TPC: total phenolic content; TFC: total flavonoid content; GAE: gallic acid equivalents; QE: quercetin equivalents.

The TPC of extracts was reported in mg GAE/g of extract. A high level of phenols was found in of all the three methanol extracts (Figure 1(a)). Therefore, HMLE had a higher phenolic content (107.41 ± 9.66 mg GAE/g of extract) compared to HMBE (93.19 ± 6.7 mg GAE/g of extract) and HMRE (68.32 ± 5.44 mg GAE/g of extract).

The TFC of extracts was expressed as mg QE/g of extract. All the extracts had high flavonoid content (Figure 1(b)). Therefore, HMLE was richer in flavonoids (53.67 ± 5.09 mg QE/g of extract) compared to HMBE (42.04 ± 4.03 mg QE/g of extract) and HMRE (24.54 ± 1.03 mg QE/g of extract).

### 3.2. Antibacterial Activity of Extracts

The antibacterial activity of extracts and IMI was assessed by determining their MICs and MBCs against one reference strain and nine MDR isolates of *P*. *aeruginosa*. The tested extracts presented a wide range of antibacterial activity, with MICs ranging from 16 to 2048 μg/mL. HMLE and bark extract were active against all the tested *P*. *aeruginosa* (100%), while root extract showed activity against 90% of the *P*. *aeruginosa* tested. All extracts showed high activity (MIC < 100 μg/mL) on at least one of the tested bacteria. *H*. *madagascariensis* leaf extract was the most active extract, with MIC of 16 μg/mL, 32 μg/mL and 64 μg/mL against *P*. *aeruginosa* ATCC27853, PA2, PA4 and PA9, respectively. The reference antibacterial (imipenem) was active on all the tested microorganisms, with MICs ranging from 4 to 256 μg/mL. Overall, the MBCs were greater than 2048 μg/mL for extracts and 256 μg/mL for IMI (Table 2). In cases where MBCs were observed (3/10), the MBC/MIC ratios were generally greater than four (Table 2).

### 3.3. Modes of Action of the Most Active Extract

#### 3.3.1. Effect of HMLE on the Cytoplasmic Membrane Integrity of *P*. *aeruginosa*

**Figure 2:**
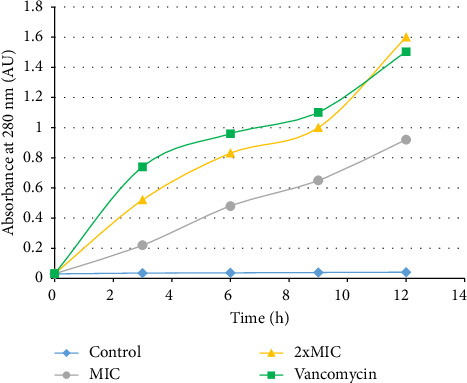
Absorbances of 260-nm absorbing materials in the supernatants of *P*. *aeruginosa* PA2 treated with HMLE. MIC: minimum inhibitory concentration. HMLE: *H*. *madagascariensis* leaf extract. The MIC of vancomycin and HMLE against *P. aeruginosa* PA2 was 32 μg/mL, respectively. Data were expressed as the mean of three replicates.

The cell release of 260-nm absorbing materials was used to analyse the integrity of the cytoplasmic membrane in treated and untreated *P*. *aeruginosa* PA2. A time-dependent cell leakage of 260-nm absorbing materials was observed in treated cells. In fact, after treatment with HMLE (MIC and 2xMIC), the absorbance of the culture increased up to 0.92 at the MIC, and 1.60 at 2xMIC. The same result was obtained with the VAN-treated *P*. *aeruginosa* PA2 (up to 1.50). In contrast, no significant change of the absorbance was observed in *P*. *aeruginosa* PA2 control cells at 260 nm until 12 h of incubation (Figure 2).

#### 3.3.2. Effect of HMLE on the Catalase Activity of *P*. *aeruginosa*

**Figure 3:**
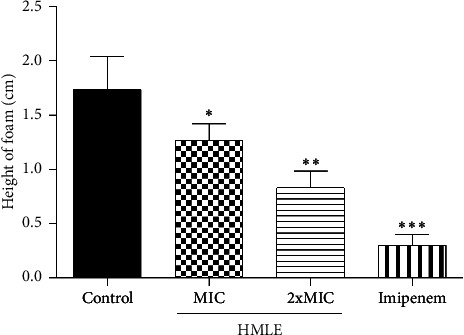
Effect of HMLE on the catalase activity of *P*. *aeruginosa* PA2. MIC: minimum inhibitory concentration; HMLE: *H. madagascariensis* leaf extract. The MIC of imipenem and HMLE against *P*. *aeruginosa* PA2 were 16 and 32 μg/mL, respectively. Data were expressed as mean ± SD of three replicates; ^∗^ (*p* < 0.05), ^∗∗^ (*p* < 0.01) and ^∗∗∗^ (*p* < 0.001).

The effect of HMLE on the catalase activity of *P*. *aeruginosa* PA2 was evaluated by comparing the amount of H_2_O_2_ remaining in the treated cells and the control. The results indicate that HMLE induced a significant decrease in catalase activity in treated *P*. *aeruginosa* PA2 (*p* < 0.05) compared to control cells. The height of foam was 1.73 ± 0.30 cm for the control cells, 1.27 ± 0.15 cm and 0.83 ± 0.15 cm for the HMLE treated cells at MIC and 2xMIC, respectively. Imipenem, used as reference antibiotic, showed the highest catalase inhibition activity (0.30 ± 0.10 cm) compared to HMLE (Figure 3).

### 3.4. Antibiotic Modulatory Effects of Extracts

In order to select the extracts likely to potentiate the antibiotic activity, a preliminary experiment carried out against *P*. *aeruginosa* (PA124) (Supporting Information, S1) allowed selection of HMLE as potential antibiotic modulator. Then, it was tested in combination with antibiotics against other MDR *P*. *aeruginosa*. The antibiotics activity against MDR isolates was improved 2- to 16-fold in the presence of HMLE at its subinhibitory concentration (MIC/8). The more representative effect was observed for the combination of HMLE with KAN, DOX, TET and VAN on PA12. The MIC decreased from 32 to ≤ 2 μg/mL for TET and DOX, and from 256 to 32 *μ*g/mL for KAN. Globally, HMLE improved the activity of KAN on 4/5 (80%), STR on 3/5 (60%), DOX, TET and VAN on 2/5 (40%) and IMI on 1/5 (20%) of the tested MDR isolates. However, no potentiation effect (0/6) was observed with CIP, CEF and AMP in the presence of HMLE (Table 3).

## 4. Discussion

Due to the rapid emergence of bacteria with MDR features, there is a shortage of new antibiotics, highlighting the urgent need to search for new antimicrobial agents. Secondary metabolites with pharmacological properties, including antimicrobial effects have been shown to be abundant in medicinal plants [8]. Moreover, many research and review papers have shown that extracts from some African medicinal plants are capable of combating bacterial infections, specifically those caused by MDR bacteria [8, 9, 33, 34]. This study was undertaken to determine the phytochemical composition, antibacterial activity, modes of action, antibiotic resistance–modifying effects of the leaf, bark and root extracts of *H*. *madagascariensis* against MDR *P*. *aeruginosa*.

Based on the cutoff values of MIC defined by Tankeo and Kuete [35], all extracts showed excellent (32 < MIC ≤ 128 μg/mL) to outstanding activities (MIC ≤ 32 μg/mL) on at least three of the tested bacteria, with HMLE as the most active extract. MBC/MIC > 4 was recorded only on 3/10 of the tested bacteria. Therefore, by carefully analysing these ratios, the tested extracts tend to present bacteriostatic effects (MBC/MIC > 4). The same finding was made by Tankeo et al. [17], who showed that the methanol extracts of bark and leaves of *H*. *madagascariensis* had strong inhibitory activities on a panel of sensitive and MDR Gram-negative bacteria, with MICs ranging from 8 to 1024 μg/mL. The high activity displayed by *H*. *madagascariensis* extracts may be due to their composition in secondary metabolites such as phenols, flavonoids and terpenoids detected in this study. Phytochemicals belonging to these chemical classes have been isolated in *H*. *madagascariensis*. Some of them are ferruginin A [17], euxanthone, harunmadagascarin D, kenganthranol [36] and astilbin [37] and could be responsible of the strong antibacterial activity obtained in this study. In fact, Tankeo et al. [17] have identified an anthranoid named ferruginin A as the active antibacterial ingredient of the bark methanol extract of *H*. *madagascariensis*, with MICs ranging from 4 to 256 μg/mL. Euxanthone, harunmadagascarin D and kenganthranol C isolated from the leaf extract of *H*. *madagascariensis* showed strong activity against *Bacillus megaterium* [36]. A flavanone named astilbin, isolated from the ethyl acetate leaf extract of *H*. *madagascariensis*, displayed strong activity against *Acinetobacter* sp., *Moraxella* sp., *Micrococcus luteus* and *Staphylococcus epidermidis* [37]. The results of this research suggest that the methanol leaf extract from *H*. *madagascariensis* may serve as a candidate for developing new antibacterial agents to treat infections caused by MDR *P*. *aeruginosa*. Studies based on the isolation and the mechanisms of actions of bioactive compounds such as ferruginin and astilbin should be envisaged.

A rise in absorbance at 260 nm in the extracellular compartment of the bacteria indicates the presence of nucleic acids or their derivatives, thus indicating a loss of membrane integrity [28]. In this study, an increase of the absorption at 260 nm was observed in HMLE-treated bacteria compared to the control, in concentration-dependant manner. These observations suggest a release of nucleic acids out of *P*. *aeruginosa* PA2 membrane, thus the contribution of HMLE to the alteration of the of *P*. *aeruginosa* PA2 membrane leading to its death. The membrane disruption action of HMLE could be attributed to lipophilic compounds such as terpenes detected in the extract [38].

Previous studies have demonstrated that mutant pathogens lacking catalase are more vulnerable to oxidative stress and assaults from the host immune response [39]. Our investigation showed that in the presence of HMLE, the catalase activity of *P*. *aeruginosa* PA2 has significantly decreased, suggesting that HMLE increases the oxidative stress in bacteria, and this may help the host immune system to eliminate the bacteria. The reduction of *P*. *aeruginosa* PA2 catalase activity by HMLE may be due to the presence of secondary metabolites such as carvacrol, and other phenolic acids which are known to act through inactivation of the bacterial antioxidant enzyme systems [29, 40].

The emergence of MDR bacteria highlights the urgent need for innovative treatment options to combat resistant bacteria. One promising approach is to combine plant extracts or isolated compounds with antibiotics [9]. In this work, we have evaluated the antibiotic modulation effects of the methanol leaves extract of *H*. *madagascariensis*. The activity of the tested antibiotics was improved 2- to 16-fold against MDR isolates in the presence of HMLE (MIC/8). MICs of TET and DOX were reduced from 32 to ≤ 2 μg/mL and that of KAN from 256 to 32 *μ*g/mL against the tested MDR *P*. *aeruginosa*. This suggests that compounds present in HMLE could act as inhibitors of efflux pumps [41] and *β*-lactamase production [42], known as examples of resistance mechanisms in *P*. *aeruginosa*.

## 5. Conclusion

This study showed that *H*. *madagascariensis* extracts contain phytochemicals such as phenols and flavonoids that are known for their antimicrobial properties. It also gave valuable insight into the potential usage of *H*. *madagascariensis* methanol extracts, specifically leaf extract (HMLE), to treat diseases triggered by MDR *P*. *aeruginosa*. Beyond its antibacterial properties, HMLE warrants development as antibiotic adjuvant for MDR infections. This work supports the medicinal properties of *H*. *madagascariensis* even though further evaluation of its remarkable properties requires in-depth investigations. For instance, the isolation and target study of the bioactive compounds from HMLE is crucial in developing appropriate antipseudomonal drugs. Moreover, the toxicity and in vivo studies will be essential to encourage its further use.

## Figures and Tables

**Table 1:** Qualitative phytochemical contents of *H*. *madagascariensis* extracts.

Phytochemical classes	Extracts		
	Leaf	Stem bark	Root
Alkaloids	+	+	+
Terpenoids	+	+	+
Saponins	+	+	+
Phenols	+	+	+
Flavonoids	+	+	+
Tannins	+	+	+
Anthocyanins	+	+	+

*Note:* (+): present; (−): absent.

**Table 2:** Antibacterial activity (MIC and MBC) of the methanol extracts of *H*. *madagascariensis* and imipenem against *Pseudomonas aeruginosa*.

*P*. *aeruginosa strains*	Samples, MIC and MBC (μg/mL)							
	Leaf		Bark		Roots		Imipenem	
	MIC	MBC	MIC	MBC	MIC	MBC	MIC	MBC
ATCC27853	**16**	256	**64**	512	**64**	1024	16	256
PA1	256	—	256	—	512	—	16	> 256
PA2	**32**	—	**64**	—	**128**	—	16	> 256
PA3	**128**	2048	256	—	256	—	16	> 256
PA4	**64**	2048	256	—	**128**	—	16	> 256
PA7	256	—	**128**	—	1024	—	4	> 256
PA9	**64**	—	256	2048	256	2048	4	> 256
PA12	**128**	—	**128**	—	512	—	128	256
PA14	1024	—	1024	—	—	—	256	> 256
PA124	1024	—	256	2048	**128**	2048	32	> 256

*Note:* —: MIC or MBC > 2048 μg/mL; values in bold represent outstanding (MIC ≤ 32 μg/mL) and excellent activities (32 ≤ MIC ≤ 128 μg/mL).

Abbreviations: MBC, minimum bactericidal concentration; MIC, minimum inhibitory concentration.

**Table 3:** MIC of the antibiotics in the presence and absence of *H. madagascariensis* leaf extract (MIC/8) against resistant *P*. *aeruginosa* isolates.

Antibiotic	HMLE concentration	Bacteria, MIC (μg/mL) and modulation factor (in brackets)					Modulation effect (%)
		PA 14	PA12	PA3	PA1	PA124	
Ciprofloxacin	0	2	≤ 0.5	≤ 2	1	≥ 32	0
	MIC/8	2 (1)	≤ 0.5 (na)	≤ 2 (na)	1 (1)	32 (≥ 1)	

Imipenem	0	256	128	16	16	32	20
	MIC/8	256 (1)	128 (1)	16 (1)	16 (1)	8 (**4**)	

Doxycycline	0	64	32	≤ 2	64	128	40
	MIC/8	64 (1)	≤ 2 (**≥ 16**)	4 (≤ 0.5)	64 (1)	16 (**8**)	

Streptomycin	0	32	≤ 2	8	32	256	60
	MIC/8	16 (**2**)	≤ 2 (na)	16 (0.5)	16 (**2**)	64 (**4**)	

Kanamycin	0	256	256	256	256	16	80
	MIC/8	64 (**4**)	32 (**8**)	32 (**8**)	64 (**4**)	16 (1)	

Tetracycline	0	256	32	16	256	128	40
	MIC/8	256 (1)	≤ 2 (≥ **16**)	16 (1)	256 (1)	64 (**2**)	

Ceftriaxone	0	32	≤ 2	8	16	—	0
	MIC/8	32 (1)	≤ 2 (na)	32 (0.25)	16 (1)	—	

Ampicillin	0	—	—	256	—	—	0
	MIC/8	—	—	512 (0.5)	—	—	

Vancomycin	0	—	4	512	—	—	40
	MIC/8	—	≤ 2 (**≥ 2**)	256 (**2**)	512 (na)	—	

*Note:* HMLE: *H*. *madagascariensis* leaf extract; —: MIC note detected up to 512 μg/mL; na: not applicable; values in bold represent modulation factors ≥ 2. %: Modulation effect in percentage for each antibiotic.

Abbreviations: MIC, minimum inhibitory concentration; PA, *Pseudomonas aeruginosa*.

## Data Availability

The data that support the findings of this study are available from the corresponding author upon reasonable request.

## Supporting Information

S1: Preliminary antibiotic-resistant modulation effects of extracts (MIC/8) against *P*. *aeruginosa* PA124.
