# Supplementary material for: Phytochemical Composition, Antibacterial Activity, Modes of Action, and Antibiotic Resistance–Modifying Effects of Harungana madagascariensis (Hypericaceae) Against Multidrug-Resistant Pseudomonas aeruginosa
Source: Scientifica (Cairo). 2025 Jul 13;2025:8950117. doi: 10.1155/sci5/8950117 (PMC12277053; doi:10.1155/sci5/8950117)
Supplement: Supporting Information — Additional supporting information can be found online in the Supporting Information section. [file 8950117.f1.pdf]

Phytochemical composition, antibacterial activity, modes of action, and antibiotic-resistance modifying effects of *Harungana madagascariensis* (Hypericaceae) against multidrug-resistant *Pseudomonas aeruginosa*

Richard Mouozong[a], Aimé Gabriel Fankam[a]*, Varelle Lambou Diffo[a], Victor Kuete[a]

[a]*Department of Biochemistry, University of Dschang, Dschang, Cameroon*

***Corresponding author:** *E-mail*: *agfankam@yahoo.fr; ORCID : http://orcid.org/0000-0001-7008-7453 (Dr. Aimé Gabriel Fankam).*

Other authors' e-mail addresses:

Richard Mouozong : mouozongrichard@gmail.com

Varelle Lambou Diffo : diffovarelle@gmail.com

Prof. Dr. Victor Kuete: kuetevictor@yahoo.fr

S1. Preliminary antibiotic-resistant modulation effects of extracts (MIC/8) against *P. aeruginosa* PA124.

ATB	Extracts' Concentration	***H. madagascariensis* (leaves)**	*H. madagascariensis* (bark)	*H. madagascariensis* (roots)
CIP	0	≥32	≥32	≥32
	CMI/8	32(nd)	≥32(nd)	≥32(nd)
IMI	0	32	32	32
	CMI/8	8(**4**)	64(0,5)	32(1)
DOX	0	128	128	128
	CMI/8	16(**8**)	256(0,5)	128(1)
STR	0	256	256	256
	CMI/8	64(**4**)	128(**2**)	128(**2**)
KAN	0	16	16	16
	CMI/8	16(1)	32(0,5)	32(0,5)
TET	0	128	128	128
	CMI/8	64(**2**)	128(1)	128(1)
CEF	0	-	-	-
	CMI/8	-	-	256(nd)
AMP	0	-	-	-
	CMI/8	-	-	-
VAN	0	-	-	-
	CMI/8	-	-	-

KAN: kanamycin; AMP: ampicillin; CEF: ceftriaxone; CIP: ciprofloxacin; DOX: doxycycline; IMI: imipenem; STR: streptomycin; TET: tetracycline; VAN: vancomycin; −: Undetectable MIC up to 512 µg/mL; (): Modulation factor; nd: not determined; Values in bold represent modulation factors ≥ 2.
